# Seasonal Influenza and Low Flu Vaccination Coverage as Important Factors Modifying the Costs and Availability of Hospital Services in Poland: A Retrospective Comparative Study

**DOI:** 10.3390/ijerph18105173

**Published:** 2021-05-13

**Authors:** Robert Susło, Piotr Pobrotyn, Lidia Brydak, Łukasz Rypicz, Urszula Grata-Borkowska, Jarosław Drobnik

**Affiliations:** 1 Gerontology Unit, Public Health Department, Wroclaw Medical University, 51-618 Wroclaw, Poland; jaroslaw.drobnik@umed.wroc.pl; 2 University Clinical Hospital Management, Wroclaw Medical University, 50-560 Wroclaw, Poland; dn@usk.wroc.pl; 3 National Institute of Public Health National Institute of Hygiene, 00-791 Warsaw, Poland; lbrydak@pzh.gov.pl; 4 Economics and Quality in Healthcare Unit, Public Health Department, Wroclaw Medical University, 51-618 Wrocław, Poland; lukasz.rypicz@umed.wroc.pl; 5 Family Medicine Unit, Family Medicine Department, Wroclaw Medical University, 51-141 Wroclaw, Poland; urszula.grata-borkowska@umed.wroc.pl

**Keywords:** medical economics, hospital economics, cost analysis, human influenza, influenza vaccines, immunization programs, anti-vaccination movement, absenteeism, sick leave

## Abstract

*Introduction***:** Influenza infection is associated with potential serious complications, increased hospitalization rates, and a higher risk of death. *Materials and Methods*: A retrospective comparative analysis of selected indicators of hospitalization from the University Hospital in Wroclaw, Poland, was carried out on patients with confirmed influenza infection in comparison to a control group randomly selected from among all other patients hospitalized on the respective wards during the 2018–2019 influenza season. *Results*: The mean laboratory testing costs for the entire hospital were 3.74-fold higher and the mean imaging test costs were 4.02-fold higher for patients with confirmed influenza than for the control group; the hospital expenses were additionally raised by the cost of antiviral therapy, which is striking when compared against the cost of a single flu vaccine. During the 2018–2019 influenza season, influenza infections among the hospital patients temporarily limited the healthcare service availability in the institution, which resulted in reduced admission rates to the departments related to internal medicine; the mean absence among the hospital staff totaled approximately 7 h per employee, despite 7.3% of the staff having been vaccinated against influenza at the hospital’s expense. *Conclusions*: There were significant differences in the hospitalization indicators between the patients with confirmed influenza and the control group, which markedly increased the hospital care costs in this multi-specialty university hospital.

## 1. Introduction

Influenza infections are associated with very frequent complications; their treatment requires inpatient care or prolongs hospitalization caused by other factors and increases costs [1]. In European countries, influenza epidemics typically affect approximately 5–15% of the population. For the regions of Central and Eastern Europe, influenza infections are associated with the hospitalization of approximately 30 additional patients per 100,000 inhabitants among those aged 65 years and older. Among these countries, Poland demonstrates the highest estimated additional costs of over 5 million EUR per year [2].

Conventional influenza therapy is mostly based on symptomatic treatment, often with over-the-counter (OTC) medications [3]. OTC treatments are ineffective in preventing flu and only relieve symptoms during an infection, with no effects on the virus itself. OTC medications advertised as “anti-flu” agents and intended for self-medication often produce inadequate effects, particularly in the case of influenza complications that are frequently too serious to be treated only symptomatically. In Poland, next-generation flu agents—i.e., neuraminidase inhibitors—were authorized in 2001 (zanamivir) and 2003 (oseltamivir) [4]. In recent years, causative flu treatments have become available, but their effectiveness depends on the time of administration following the infection diagnosis. The initiation of therapy does not require a previous laboratory confirmation of influenza infection. Neuraminidase inhibitors reduce the severity and duration of flu symptoms, the number of hospitalizations required, and the risk of both influenza complications and flu-related death [5]. In Poland, oseltamivir and its generic equivalents (bioequivalent to the reference agent) that were granted marketing authorization by the European Medicines Agency have been available since 2016 [6].

In contrast, an easy-to-use, safe, and relatively inexpensive method of primary prevention—i.e., a vaccine—has been available for many years. It has been shown that vaccines provided to Spanish citizens aged 65 and older from 2013 to 2015 considerably reduced the risk of hospitalization in the intensive care unit, mechanical ventilation, secondary lung infection, and a higher dependence level. As a result, the mean hospital care cost for elderly patients with influenza who had been vaccinated at least 15 days prior to hospitalization was 2.75% lower than that of patients who developed flu, despite having been vaccinated [7]. According to studies conducted in the USA, vaccination costs regarding patients aged 65 and older incurred by healthcare institutions are recovered as savings on direct hospital care costs—up to 40% of which can be avoided when vaccinated patients are compared to unvaccinated ones—with additional indirect benefits such as avoiding discomfort, disability, or lost earnings [8]. Therefore, it is no surprise that as early as 25 years ago, flu vaccination programs were proposed, especially for the whole elderly population [9]. Socioeconomic factors, such as living in an urban area and having a member of the family who has already been vaccinated, increase the willingness to vaccinate among Poles, as well as a younger age and having comorbidities [10]. Unfortunately, although the proportion of vaccinated people in Poland grows slightly every year, it remains very low (approximately 4%), especially in comparison to the values for other European countries [11]. Data concerning flu vaccination, especially coverage rates, in the countries of the European Union are highly inconsistent and incomplete. For the flu season of 2017/2018, the general population coverage rate is known only for Latvia (2.0%) and Romania (5.2%); in the previous flu season, it reached 8.4% in Lithuania. The influenza vaccination coverage rate for all healthcare workers was highest in the U.K. country of Wales, where it reached 54.7% [12]. An additional problem is the anti-vaccination movement, which is actively trying to hamper vaccine popularization [13].

Influenza infections do not spare medical staff, a fact that creates a risk of virus transmission to patients, increases absence due to illness, and increases the work burden on the other staff members in healthcare institutions. All of this leads to significant measurable economic costs [14]. Vaccination is an effective method for a nearly 90% reduction in morbidity among medical personnel, as well as a decrease of approximately 50% in symptom severity and disease duration, resulting in lower rates of absenteeism [15]. Although every dollar invested in an influenza vaccination program for healthcare workers saves employers, on average, approximately 2.5 dollars, the actual rates of influenza immunization among healthcare workers differ significantly from country to country, ranging from 2% to 60%, which is unsatisfactory. In general, they tend to improve significantly when workers are explicitly offered vaccinations, especially in cases where free-of-charge vaccinations provided by the employer are combined with an educational program addressing misconceptions about vaccinations, some incentives are offered to workers participating in vaccination programs, or participation is made compulsory as a condition of employment [16].

The elevated costs related to influenza for a hospital can be patient-, staff-, and throughput-related. Occurrences of influenza among hospitalized patients may demand, among other things, expensive additional diagnostic tests or flu-specific treatments. Occurrences of influenza among hospital staff—or their families—may cause increased absenteeism, forcing other staff members to work overtime for increased pay or even necessitating employing additional staff members to cover for those absent because of the flu, which is troublesome and expensive due to the notorious shortage of medical workforce in the labor market. Influenza may also limit the availability of hospital beds and thus impair hospital throughput, with subsequent financial losses due to the unsatisfactory level of medical service contract fulfillment. This may result from many overlapping causes, as it may require prolonging the hospitalization of patients suffering from the flu or its complications, leaving some of the available hospital beds empty due to flu patient isolation regimes, temporarily suspending hospital admissions for the duration of flu outbreaks, or even demanding the evacuation of whole hospital wards because of staff shortages. All of these negative phenomena are caused by flu among patients and hospital staff, while influenza cases could largely be effectively prevented if staff members and patients were vaccinated.

This study’s objectives were to provide evidence of the financial effectiveness of the influenza vaccination of both hospital staff and patients based on some selected parameters: Determining both the differences in laboratory testing and imaging costs between patients with confirmed influenza infection and a control group (without confirmed influenza) and the scale of the negative effects of hospital influenza outbreaks and flu season absenteeism among healthcare workers on the availability of inpatient care in a multispecialty University Hospital in Poland in comparison to the cost of vaccinating hospital staff.

## 2. Materials and Methods

In the first quarter of 2019 (during the 2018–2019 influenza season), 212 influenza cases in total were confirmed across all departments of the University Hospital in Wrocław, with 310 cases of suspected influenza infection that were monitored for flu and ultimately verified as negative. Based on the medical records and pharmacoeconomic data on the University Hospital units located at 213 Borowska Street, a comparative analysis of the hospitalization indicators was conducted for 130 patients with confirmed influenza treated during their hospital stay and for a control group of 260 individuals without a diagnosis of influenza infection. The control group was comprised of randomly selected patients from among the other people hospitalized in the departments where the influenza cases were identified between 1 January 2019 and 31 March 2019. The size of the control group in each department was determined by rounding up the number of influenza cases to the nearest ten. The random selection was performed using the pseudorandom number generator available in an Excel 2013 spreadsheet (Microsoft, Redmond, WA, USA). The costs of the laboratory tests and imaging were analyzed. All statistical analyses were performed using the data analysis software system Statistica 13.3 PL (2017, TIBCO Software Inc., Palo Alto, CA, USA). The threshold for the statistical significance of differences between the groups was set at *p* < 0.05. When, based on the Shapiro–Wilk test results, the hypothesis concerning the normalcy of the distribution of the analyzed data was rejected, further statistical analyses included the Mann–Whitney *U*-test, which is a non-parametric test for independent variables.

## 3. Results

The presentation of the results is divided into the pooled analysis of the studied parameters, followed by differences in the parameters studied between hospital departments and the costs of the laboratory testing and imaging tests. In addition, the number of admissions during the season and data on sickness absence among the hospital staff during the epidemic flu season are also presented.

### 3.1. Pooled Analysis of the Studied Parameters

During the study period (the first quarter of 2019), statistically significant differences in the costs of laboratory tests and imaging were observed between the patients with confirmed influenza infection and the control group (Table 1). Age differences between the groups were not statistically significant for any hospital department with a sufficient number of influenza cases for analyzing the statistical significance of the indicators under study.

### 3.2. Differences in the Parameters Being Studied between Hospital Departments

Considering data for the entire hospital, statistically significant differences between the study group and the control group were confirmed for the costs of laboratory tests and imaging. The data analysis for individual departments showed that among the 12 hospital departments where the number of influenza cases was sufficient to perform an analysis of statistical significance for the investigated parameters, statistically significant differences in at least one parameter were observed in the following nine departments: The Department of Anesthesiology and Intensive Therapy; the Department of Angiology, Hypertension and Diabetology; the Department of Cardiology; the Department of Internal and Occupational Diseases; the Department of Gastroenterology; the Department of Nephrology; the Department of Neurology; the Department of Rheumatology and Internal Medicine; the Department of Transplantation. The only three exceptions where such differences were not confirmed were the Department of Gynecology and Obstetrics, the Department of Ophthalmology, and the Emergency Department (Table 2).

### 3.3. Costs of Laboratory Testing

**Figure 1:**
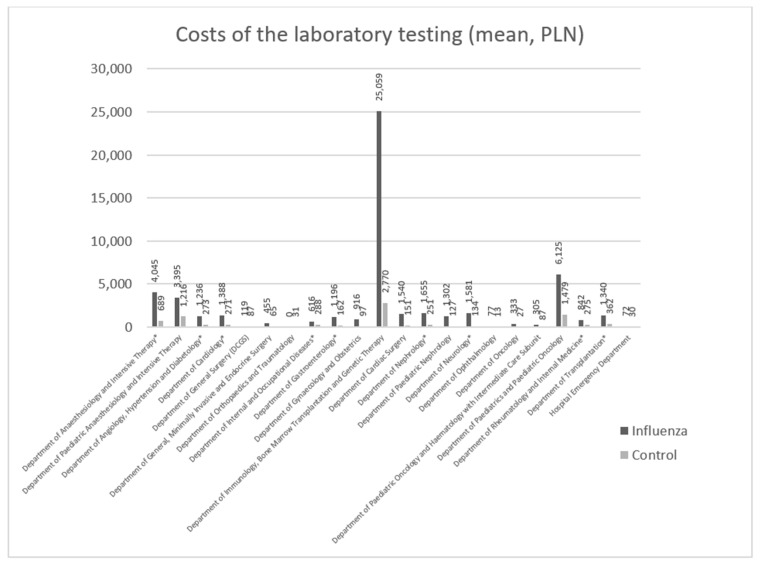
Mean costs of laboratory testing for the patients with confirmed influenza infection and for the control group, by hospital department (PLN) (1 PLN = 0.22 EUR). The departments with statistically significant differences between the groups (*p* < 0.05) are marked with asterisks (*).

For the mean cost of laboratory testing, a 3.74-fold difference was observed between the group of patients with confirmed influenza infection (1435.54 PLN; approximately 315.82 EUR) and the control group (383.42 PLN; approximately 84.35 EUR). The differences in the mean costs of laboratory testing between the groups are presented in Figure 1. The number of patients was sufficient to perform statistical analyses of the 12 departments. Statistically significant differences in the costs of laboratory testing were observed in the following nine departments: The Department of Anesthesiology and Intensive Therapy; the Department of Angiology, Hypertension and Diabetology; the Department of Cardiology; the Department of Internal and Occupational Diseases; the Department of Gastroenterology; the Department of Nephrology; the Department of Neurology; the Department of Rheumatology and Internal Medicine; the Department of Transplantation. Non-statistically significant differences were reported for only three units: The Department of Gynecology and Obstetrics, the Department of Ophthalmology, and the Emergency Department.

The largest statistically significant differences in the mean costs of laboratory testing between the patients with confirmed influenza infection and the control group were observed in the Department of Neurology, the Department of Gastroenterology, the Department of Nephrology, and the Department of Anesthesiology and Intensive Therapy. For patients with confirmed flu, they were 11.7-, 7.37-, 6.58-, and 5.87-fold higher, respectively, than for the control group. The smallest differences were observed for the Department of Internal and Occupational Diseases; the Department of Rheumatology and Internal Medicine; the Department of Transplantation; the Department of Angiology, Hypertension and Diabetology; the Department of Cardiology (2.14-, 3.06-, 3.70-, 4.52-, and 5.12-fold higher testing costs, respectively, among the patients with confirmed influenza infection (Figure 1).

The differences in laboratory testing costs between the patients with confirmed influenza infection and the control group are even more distinctly illustrated by comparisons of the minimum and maximum costs and the medians calculated for the individual hospital departments.

For the Department of Neurology, the median cost of laboratory testing was 15.3-fold higher among the patients with confirmed influenza infection than in the control group. For all nine departments in the analysis, there were situations in the control group when no cost-generating laboratory tests were performed, while for the patients with confirmed influenza infection, there were only two such units: The Department of Gastroenterology and the Department of Internal and Occupational Diseases. For the Department of Gastroenterology, the maximum cost of laboratory testing was 6.6-fold higher in the patients with flu than in the control group.

### 3.4. Costs of Imaging Tests

**Figure 2:**
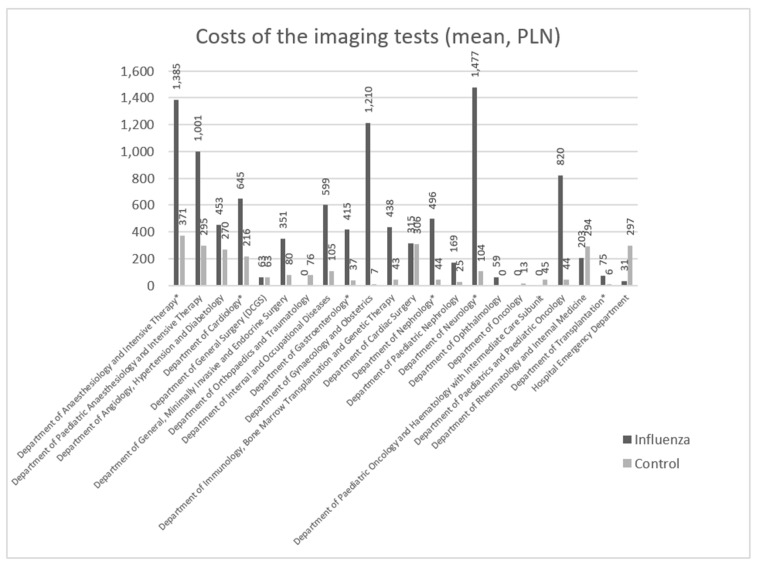
Mean costs of imaging tests for the patients with confirmed influenza infection and for the control group, by hospital department (PLN) (1 PLN = 0.22 EUR). The departments with statistically significant differences between the groups (*p* < 0.05) are marked with asterisks (*).

The mean cost of imaging tests among the patients with confirmed influenza infection (514.02 PLN; approximately 113.08 EUR) was markedly (4.02-fold) higher than in the control group (127.72 PLN; approximately 28.10 EUR). The differences in the mean costs of imaging tests between the groups are presented in Figure 2. Among the hospital units with a sufficient number of patients for statistical analysis, statistically significant differences in the costs of imaging tests were observed for the following six departments: The Department of Anesthesiology and Intensive Therapy, the Department of Cardiology, the Department of Gastroenterology, the Department of Nephrology, the Department of Neurology, and the Department of Transplantation. Statistically significant differences were not reported for the other six units: The Department of Angiology, Hypertension and Diabetology; the Department and Clinic of Internal and Occupational Diseases; the Department of Ophthalmology; the Department and Clinic of Rheumatology and Internal Medicine; the Emergency Department.

The largest statistically significant differences in the mean costs of imaging tests between the patients with confirmed influenza and the control group were observed in the Department of Neurology, the Department of Transplantation, the Department of Nephrology, and the Department of Gastroenterology. For the patients with flu, they were 14.19-, 11.93-, 11.35-, and 11.08-fold higher, respectively, than for the control group. The smallest differences were reported for the Department of Cardiology and the Department of Anesthesiology and Intensive Therapy (2.99- and 3.74-fold higher testing costs among the patients with confirmed influenza infection, respectively; Figure 2).

The differences in imaging testing costs between the patients with confirmed influenza infection and the control group are even more distinctly illustrated by comparisons of the minimum and maximum costs and the medians calculated for the individual hospital departments. Among all the patients in the control groups, imaging tests were only performed on individual patients in four departments, while there were no such departments in the case of patients with confirmed influenza infection. In all six departments in the statistical analysis, there were situations in the control group when no cost-generating imaging tests were performed, while for the patients with confirmed influenza infection there were only three such units: The Department of Cardiology, the Department of Gastroenterology, and the Department of Nephrology. For the Department of Gastroenterology, the maximum cost of imaging tests was 11.1-fold higher for the patients with flu than for the control group.

### 3.5. Number of Admissions during the Flu Period in the First Quarter of 2019 (2018–2019 Flu Season)

Over the period of increasing influenza rates throughout the 2018–2019 flu season, during the first quarter of 2019 it was necessary to forbid visiting across all hospital premises for eight weeks (22 January to 17 March 2019). Moreover, elective admissions were temporarily restricted for 10–16 days in the departments with the largest numbers of confirmed influenza cases (the Department of Cardiology, the Department of Nephrology, the Department of Gastroenterology, the Department of Internal and Occupational Diseases, and the Department of Rheumatology) or where flu outbreaks were identified (the Department of Cardiology and the Department of Nephrology). For the same reasons, the suspension of elective admissions was considered at the Department of Pediatric Hematology and the Department of Anesthesiology and Intensive Therapy, but the idea was ultimately abandoned due to the lack of alternative institutions in the region. Instead, staff members of the other hospital units were assigned to work in the affected departments in order to ensure the better isolation and care of the patients.

In the majority of the affected departments, these procedures resulted in fewer hospitalized patients if compared year-on-year to the previous flu season, when there were fewer patients with confirmed influenza infection. In the first quarter of the year 2019, which was the peak of the flu season 2018–2019, there were 22.83% fewer hospital admissions reported in the Department of Cardiology than one year before (561 vs. 727 admissions). In the case of the Department of Internal Occupational Diseases, there were 9.10% fewer hospitalizations in January, February, and March 2019 than in the respective months in 2018 (649 vs. 717 admissions); in case of the Department of Nephrology, there were 2.75% fewer hospital admissions (708 vs. 728 admissions).

### 3.6. Sickness Absence among the Hospital Staff during the Epidemic Flu Season

Over the period preceding the 2018–2019 flu season, the staff members received the flu vaccine at the hospital’s expense for the first time; overall, 297 employees were vaccinated (7.3% of the entire hospital staff). The number of employees who were vaccinated outside the hospital is unknown.

The absenteeism among the hospital staff in the first quarter of 2019—i.e., at the peak of the 2018–2019 flu season, when there were more patients with confirmed influenza infection than a year before—increased by 16.04% compared to the same period of the previous flu season (2017–2018), which corresponds to a significant increase of more than one day in the mean time of absence per employee (Table 3).

The influence of influenza on staff absenteeism is clearly visible, as in the first quarter of the year 2019, when there were more patients with confirmed influenza than in the year before, the rate of absenteeism was 10.01% higher than that for an average quarter of the year, calculated for the whole 2018–2019 flu season (from 1 October 2018 to 30 September 2019). This was the opposite in the case of the 2017–2018 flu season, when there were fewer patients with confirmed influenza and the staff absenteeism in the months from January to March 2018 was 13.70% lower in the whole 2017–2018 flu season—i.e., from 1 October 2017 to 30 September 2018 (Table 4)—than in an average quarter of the year.

**Figure 3:**
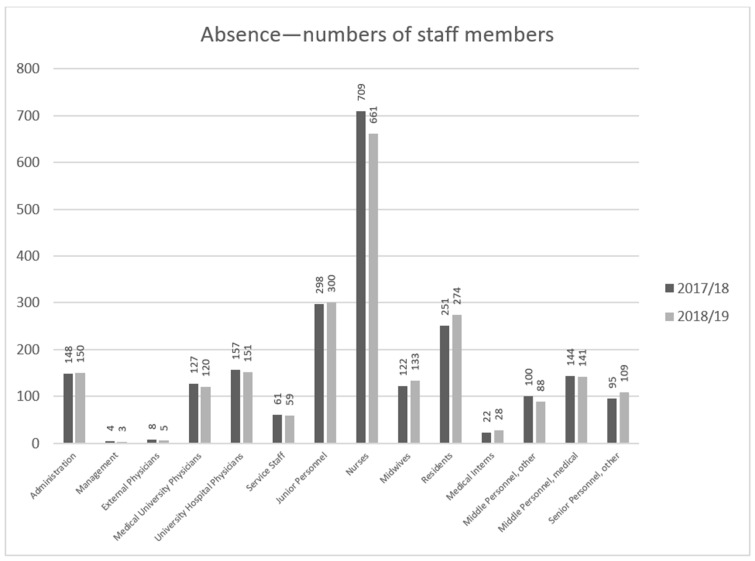
Hospital employees with reported absence due to illness during the 2017–2018 and 2018–2019 flu seasons, by healthcare professional group.

**Figure 4:**
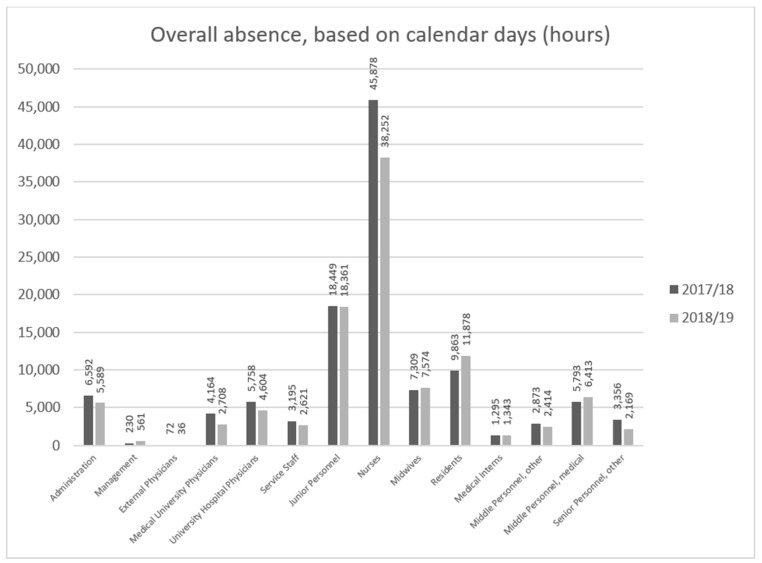
Overall lost working days among the hospital employees with absence due to illness reported during the 2017–2018 and 2018–2019 flu seasons, by healthcare professional group.

Throughout the 2017–2018 flu season, the absence of 2246 employees in total among hospital personnel was reported, while the overall number of staff members on sick leave during the 2018–2019 season amounted to 2222 employees. The largest number of infections was observed among the nursing staff (Figure 3), which also meant the largest loss of working hours (Figure 4).

**Figure 5:**
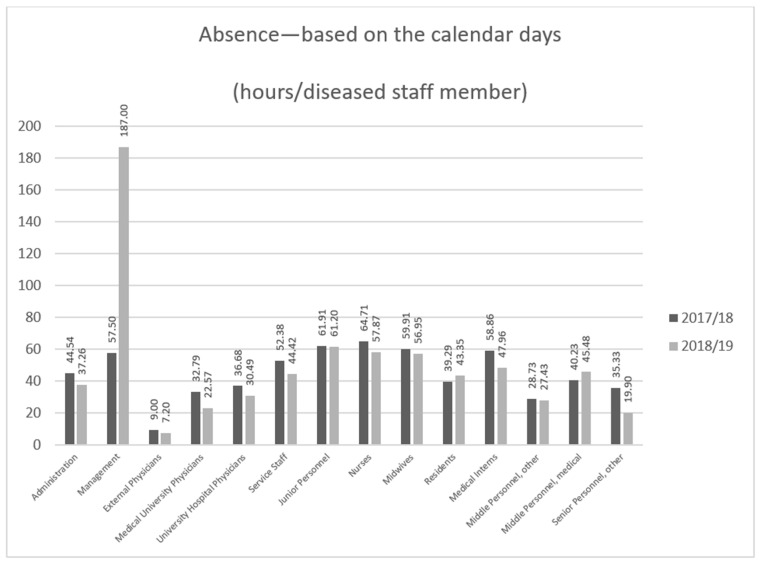
Mean lost working days among hospital employees with absence due to illness reported during the 2017–2018 and 2018–2019 flu seasons, by healthcare professional group.

However, the largest loss of working hours among nurses was not the result of the longer flu duration among the nursing staff compared to among other occupational groups (Figure 5).

## 4. Discussion

According to the literature evidence, influenza is responsible for 30% of the total annual burden of the 31 most common infectious diseases in Europe [17], as influenza virus infections each year cause up to 1.8–3.5 additional hospitalizations per 10,000 persons, with an average cost of 6100–8300 EUR [18]. Influenza infections not only increase the risk of required hospitalization, but also prolong its duration (which was also demonstrated in the previously analyzed data from the University Hospital in Wrocław) and raise the overall cost of treatment [1]. The analysis of data concerning the University Hospital in Wrocław confirmed this. This study revealed statistically significant differences in the costs of laboratory tests and imaging between patients with confirmed influenza infection and a control group. The cost of a single diagnostic test, especially for an imaging test, is high and this causes the total cost of diagnostics to rise dramatically with each consecutive test the patient has. This leads to large differences in the costs associated with the hospitalization of a patient who is subjected to no diagnostic tests in comparison to another patient who has several; this explains the large standard deviation values we found (Table 1). Compared to the control group, the mean cost of laboratory testing was over 3.5-fold higher and the cost of imaging tests was over 4.0-fold higher in the group of patients with confirmed influenza. In this context, it should be emphasized that the additional direct cost incurred by the hospital for treating the patients with confirmed influenza infection was the cost of antiviral therapy—administering oseltamivir (Tamiflu^®^) to all 130 patients. The treatment cost per patient amounted to 117.19 PLN (approximately 25.78 EUR), which means that the antiviral therapy itself generated an additional necessary expense of 15,234.19 PLN (approximately 3351.52 EUR) for the hospital. By comparison, the retail price of a modern quadrivalent influenza vaccine—Sanofi Vaxigrip Tetra™—was 45 PLN (approximately 9.9 EUR) before the 2018–2019 flu season, and patients aged 65 and older were entitled to a 50% reimbursement covered by the National Health Fund [19]. Consequently, the cost of specific flu antiviral therapy of one patient alone could cover the cost of vaccinating approximately three people.

Not all departments of the University Hospital in Wrocław had sufficient numbers of hospitalized influenza patients to demonstrate statistically significant differences in comparison to the control group at the chosen significance level. In all of the departments where analysis could be performed, statistically significant differences in at least one of the indicators in question were observed between the two groups, except for the Department of Ophthalmology, the Emergency Department, and the Department of Gynecology and Obstetrics. The lack of such differences is particularly surprising in the case of the last unit, since pregnant women—especially during the last trimester—are at a higher risk of severe disease course and serious complications [20].

In the departments with statistically significant differences in the costs of laboratory testing, the mean costs of these tests were markedly higher (2.14- to 11.78-fold) among the patients with confirmed influenza in comparison with the control group (Figure 1), which is consistent with the requirement for extended diagnosis due to flu complications, as described in the literature. Even more pronounced (over 10-fold) differences, which are unfavorable to patients with confirmed influenza infection, were demonstrated in comparisons of the median, minimum, and maximum costs of laboratory testing between the two groups.

The data from the departments with statistically significant differences in imaging costs show that the typical mean costs of these tests were markedly higher (2.99- to 14.19-fold) among the patients with confirmed influenza than in the control group (Figure 2), which is consistent with the requirement of extended diagnosis of influenza or its complications found in the literature. In some hospital departments, imaging tests were only performed on patients with influenza infection, being unnecessary for the control group.

These considerations lead to the conclusion that there are significant differences in the key indicators of hospitalization between the patients with confirmed influenza and the control group that markedly increase hospital care costs in this Polish multispecialty University Hospital. It should be emphasized that the increased hospitalization costs attributed to patients with influenza are not in any way reflected by the system of healthcare service financing by the public payer, the National Health Fund. The influenza vaccinations are not reimbursed for every Polish citizen either [19], while it is known that the vaccination of schoolchildren is crucial in preventing influenza transmission in all age groups, as it determines the level of herd immunity to influenza and eliminates children as the key drivers of influenza transmission [21].

Particularly large numbers of influenza cases or flu outbreaks in hospital departments, especially those related to internal medicine, required the restriction or temporary suspension of elective admissions, which resulted in significantly less access to healthcare services for the population of Lower Silesia. Consequently, influenza infections among the hospital patients limited the healthcare service availability for several weeks, resulting in reduced admission rates in the departments related to internal medicine during the 2018–2019 influenza season when compared year-on-year to the previous season. Moreover, the inconvenience of the nearly two-month-long ban for patients and their relatives visiting due to the increased numbers of influenza infections should be emphasized. In this context, it needs to be emphasized that universal vaccination is a cost-effective measure to avoid influenza outbreaks in institutions; assuming that all people get vaccinated, the total healthcare costs will be reduced by approximately half, while half of the remaining healthcare cost is related to vaccinations [22].

At the peak of the 2018–2019 flu season, in the first quarter of 2019, the absence due to illness among the personnel at the University Hospital in Wrocław was over one day longer on average than during the same period of the previous year, reaching a mean of 7 h per employee over a three-month period (Table 3). Over a 12-month period, the absence of staff due to illness during the 2018–2019 flu season amounted to 25.5 h on average, which was 1.5 h less than that during the previous season (Table 4). The above data suggest that influenza infection is an important factor that affects absenteeism among hospital medical personnel, even though one can expect that medical professionals effectively employ protective measures, including flu symptom vigilance and early voluntary self-isolation [23]. This happened despite a wide informational campaign related to the legitimacy of a flu vaccination program for staff, which was conducted in the hospital before the 2018–2019 flu season, and despite 7.3% of the personnel having been vaccinated at the employer’s expense. Although far too low to positively influence the epidemiological situation in the hospital, that indicator can be considered a success as it was better that the average as, according to ECDC data, the proportion of vaccinated healthcare professionals in Poland is as low as 6.4%. This very low proportion of vaccinated healthcare professionals is evidence of the lack of awareness—not only of the risks associated with influenza infection and its economic consequences for the whole country, but also of the risk of healthcare professionals transmitting the infection to others. Raising awareness related to the risks of influenza [24] reinforced by proper education is crucial in order to achieve a positive attitude toward influenza immunization [25]. On the contrary, the rate of immunization is low, even in the case of Polish medical students who are future healthcare workers, although they have good knowledge of the flu vaccine and demonstrate positive attitudes toward vaccines [26]. This phenomenon is common for many European countries, where the majority medical students and medical doctors declare very positive general attitudes toward vaccinations, but they rarely support them with behaviors and vaccinate themselves [27]. The literature data suggest that in cases of healthcare workers, multiple factors need to be dealt with in this context, including professional duty and ethics, the need for self-determination, vaccine hesitance, or even conscientious refusal [28]. The higher staff absenteeism due to illness led to organizational problems, a heavier work burden on the remaining employees during a challenging period (resulting from additional requirements for more isolation and care of patients with confirmed influenza). This also resulted in additional costs associated with overtime payment or hiring replacement staff, which was twice as difficult in light of the widespread scarcity of medical and support staff on the job market. This particularly applies to nursing staff, who make up the highest numbers of flu cases (Figure 3, Figure 4 and Figure 5).

The exact cause of work absenteeism is not revealed to the employer in Poland due to privacy reasons, so it was impossible to determine which absences among hospital staff were linked to influenza; however, one can make rough extrapolations based on the absenteeism statistics for the whole country. Pre-school and schoolchildren, higher education participants, people living with disabilities that make them unemployable, and people who have retired already neither work at hospitals nor require sick leave registered by Polish state statistics. On the contrary, people who belong to all other groups and whose illness-related absenteeism demands official reporting are employed both generally statewide and specifically by hospitals. In the year 2019, respiratory tract infections were responsible for 12.3% of all reported days of absence among the workforce due to illness, which was equivalent to above 5.24 million issued sick leave certificates; the average length of such an absence was 5.57 days [29]. In the 2018/2019 flu season, there were above 3.47 million flu and flu-like infection cases reported in Poland [30]. This means that flu and flu-like infections were responsible for approximately 66% of all reported cases of respiratory tract infections and—assuming that the duration of an average influenza infection is similar to the average length of absence due to respiratory tract infections—this translates into approximately 8.2% of all reported days of absence among the workforce in Poland. Assuming that the structure of absence causes in the University Clinical Hospital in Wrocław did not significantly differ from the structure typical for the general Polish population, flu and flu-like infections were responsible, depending on the analyzed flu season, for approximately 8570.90–9415.81 h of hospital staff absence. In Poland, during the year 2019, the total cost of employing an employee at the minimal hourly rate was 17.75 PLN (approximately 3.91 EUR) [31]. Consequently, the minimal cost of finding a replacement for workers suffering from flu or flu-like illnesses would be no less than approximately 152,133.48–167,130.63 PLN (approximately 33,469.77–36,769.18 EUR), depending on the given flu season. The true replacement cost burden for clinical hospitals is, in fact, several times bigger than the rough estimations above, as healthcare workers, as a qualified workforce, do not work for minimal hourly rates and the pay for working overtime is significantly higher than the standard pay. In contrast, assuming the epidemiologically satisfying target level of 85% participation, one seasonal workforce flu vaccination program at the University Hospital in Wrocław would translate into 3400 doses of vaccine at a retail price of 45 PLN (approximately 9.9 EUR) a dose [19] and, in total, a cost of approximately 153,000.00 PLN (approximately 33,660.40 EUR) covered in full by the employer. The actual cost of the program for a hospital is likely to be much lower than the estimation above, as large batches of vaccines are available at discounted wholesale prices. It is obvious that enforcing vaccinations of the entire hospital staff where the cost is covered in full by the employer would be financially beneficial, as the cost of the vaccines is balanced by the avoided losses resulting from absenteeism alone.

## 5. Conclusions

The results of this study confirm that the costs related to influenza are an important issue to consider in public health measures, demanding increasing efforts to popularize flu vaccinations.

Flu cases in patients who stay at a hospital increase hospitalization costs, including significantly greater expenses on laboratory testing (3.74-fold) and imaging tests (4.02-fold) and demand for additional spending on specific antiviral therapy. Therefore, the increased hospitalization costs attributed to patients with influenza need to be reflected by the system of healthcare service financing by the public payer, the National Health Fund.

Even a single case of influenza may cause limitations in the availability of in-patient services, as rooms with multiple beds often cannot be fully utilized because of flu patient isolation regime requirements. In cases of flu outbreaks, whole departments are forced to suspend patient admissions for weeks. Flu among hospital staff causes increased absenteeism from work. Consequently, admissions to the hospital may be temporarily limited or even fully suspended because of the flu due to the lack of available free hospital beds or the hospital staff deficit.

This study demonstrated that the losses resulting from flu among hospital staff alone are disproportionately large in comparison to the cost of flu vaccinations; thus, it is financially profitable for the employer to provide free-of-cost influenza vaccinations to the entire workforce of the hospital. The necessary actions taken in order to reduce the effects of seasonal influenza infections on hospital operations involves providing obligatory flu vaccinations to all staff members at the hospital’s expense and increased alertness to ensure the early identification and isolation of patients with suspected influenza infection, as well as the routine postponement of elective hospital admissions for patients without documented flu vaccination. The high social costs resulting from flu-related limitations in hospital service availability to patients add to the long list of arguments that support introducing flu vaccine reimbursement covered by the National Health Found for all citizens who are willing to vaccinate themselves.

The results of this study add evidence and scale to the—common but still scarcely supported by measurable evidence—knowledge that patients ill with influenza cause a significant rise in hospital treatment costs, allowing the negotiation of increased reimbursement for treating such patients more effectively. The presented results also objectively support the vital public health mission to popularize further flu vaccination, especially among the staff employed at medical facilities.

The scope of this study was limited to the analysis of laboratory testing and radiology test costs in one hospital during the flu season of 2018/2019; staff absenteeism and the availability of hospital services were analyzed for the flu seasons of 2017/2018 and 2018/2019. The authors are planning further analyses, including analyses of data from more hospitals and concerning more flu seasons—especially the influenza seasons to come, as a higher flu vaccination coverage is expected—in order to monitor its effects.

## Figures and Tables

**Table 1:** The main hospitalization indicators for the hospital patients with confirmed influenza infection and for the control group (1 PLN = 0.22 EUR).

Variable	Patients with Confirmed Influenza Infection (*N* = 130)			Control Group (*N* = 260)			Test Result
	Mean	Median	Standard Deviation	Mean	Median	Standard Deviation	
Cost of laboratory testing [PLN]	1435.54	908.80	2480.29	383.42	102.72	1544.09	*p* < 0.05
Cost of imaging [PLN]	514.02	202.37	782.95	127.71	0.00	337.00	*p* < 0.05

**Table 2:** Number of influenza and control group patients. The departments with statistically significant differences in their costs for laboratory testing and costs for imaging tests between the groups numerous enough for the statistics to be significant (*p* < 0.05) are marked with asterisks (*).

Hospital Department	Influenza Patients	Control Group Patients	Statistically Significant Differences in Costs of the Laboratory Testing	Statistically Significant Differences in Costs of the Imaging Tests
Department of Anaesthesiology and Intensive Therapy	6	10	*	*
Department of Paediatric Anaesthesiology and Intensive Therapy	1	10		
Department of Angiology, Hypertension and Diabetology	5	10	*	
Department of Cardiology	27	30	*	*
Department of General Surgery	1	10		
Department of General, Minimally Invasive and Endocrine Surgery	1	10		
Department of Orthopaedics and Traumatology	1	10		
Department of Internal and Occupational Diseases	18	20	*	
Department of Gastroenterology	10	10	*	*
Department of Gynaecology and Obstetrics	2	10		
Department of Immunology, Bone Marrow Transplantation, and Genetic Therapy	1	10		
Department of Cardiac Surgery	1	10		
Department of Nephrology	19	20	*	*
Department of Paediatric Nephrology	1	10		
Department of Neurology	4	10	*	*
Department of Ophthalmology	4	10		
Department of Oncology	1	10		
Department of Paediatric Oncology and Haematology with Intermediate Care Subunit	1	10		
Department of Paediatrics and Paediatric Oncology	1	10		
Department of Rheumatology and Internal Medicine	10	10	*	
Department of Transplantation	6	10	*	*
Hospital Emergency Department	9	10		
Total	130	260		

**Table 3:** Absence due to illness among the hospital staff during the peaks of the 2017–2018 and 2018–2019 flu seasons.

Epidemic Season (Peak)	Absence Due to Illness (h)	Number of Hospital Employees	Individual Absenteeism Rate (Lost Working Hours/Employee)
2017–2018	24,774	4157	5.9595
2018–2019	28,747	4093	7.0235
Average			6.4874

**Table 4:** Absence due to illness among hospital staff during the entire 2017–2018 and 2018–2019 flu seasons (calendar days).

Flu Season (Overall)	Absence Due to Illness (h)	Number of Hospital Employees	Individual Absenteeism Rate during the Entire Flu Season (Lost Working Hours/Employee)	Average Quarterly Absenteeism Rate during the flu Season (Lost Working Hours/Employee)
2017–2018	114,827	4157	27.6226	6.9057
2018–2019	104,523	4093	25.5370	6.3843
Average			26.5879	6.6470

## Data Availability

The data presented in this study are available on request from the corresponding author.
